# Autoimmune and Paraneoplastic Chorea: A Review of the Literature

**DOI:** 10.3389/fneur.2022.829076

**Published:** 2022-03-18

**Authors:** Kevin Kyle, Yvette Bordelon, Nagagopal Venna, Jenny Linnoila

**Affiliations:** ^1^Department of Neurology, Massachusetts General Hospital, Harvard Medical School, Boston, MA, United States; ^2^Department of Neurology, David Geffen School of Medicine, University of California, Los Angeles, Los Angeles, CA, United States

**Keywords:** autoimmune, paraneoplastic, chorea, diagnosis, management

## Abstract

Autoimmune chorea syndromes represent a vast array of paraneoplastic, parainfectious and idiopathic disorders. It is increasingly apparent that familiarity with these disorders is critically important, as they may be treatable or may be part of a syndrome requiring further work-up and monitoring. These disorders are mediated by an aberrant immunologic attack with resultant neuronal dysfunction, manifesting as chorea. These conditions are typically accompanied by other neurologic or systemic manifestations. In this review we outline the clinical features, epidemiologic factors, and delineate the specific antibodies associated with each of these autoimmune mediated disorders. We highlight up to date information regarding this heterogeneous group of disorders, including a discussion of parainfectious Sydenham's chorea; paraneoplastic syndromes associated with CRMP-5 (collapsin response mediated protein-5/CV2) and ANNA-1 (antineuronal nuclear antibody / Hu) antibodies, in addition to neuronal antibody-associated disorders including anti-NMDAR, LGI1 (leucine-rich glioma inactivated-1) and CASPR2 (contactin associated protein-2). We discuss the more recently described entities of IgLON5, which has evidence of both immunologic and degenerative pathophysiology, in addition to PDE-10A antibody-associated chorea. We also outline chorea secondary to systemic diseases including Systemic Lupus Erythematosus (SLE) and Primary Antiphospholipid Syndrome (PAPS). We provide a framework for diagnosis and treatment.

## Introduction

Autoimmune neurology is an evolving field with an expanding list of syndromes and antibody associations which are increasingly recognized in clinical practice. Autoimmune-mediated movement disorders are not as well-recognized, leading us to provide this update and review. Chorea, in particular, is associated with an array of autoantibodies and is a feature of some autoimmune syndromes. It has been suggested that autoimmune chorea is one of the leading causes of adult onset chorea behind Huntington's Disease (HD) and vascular etiologies (1, 2).

**Figure 1:**
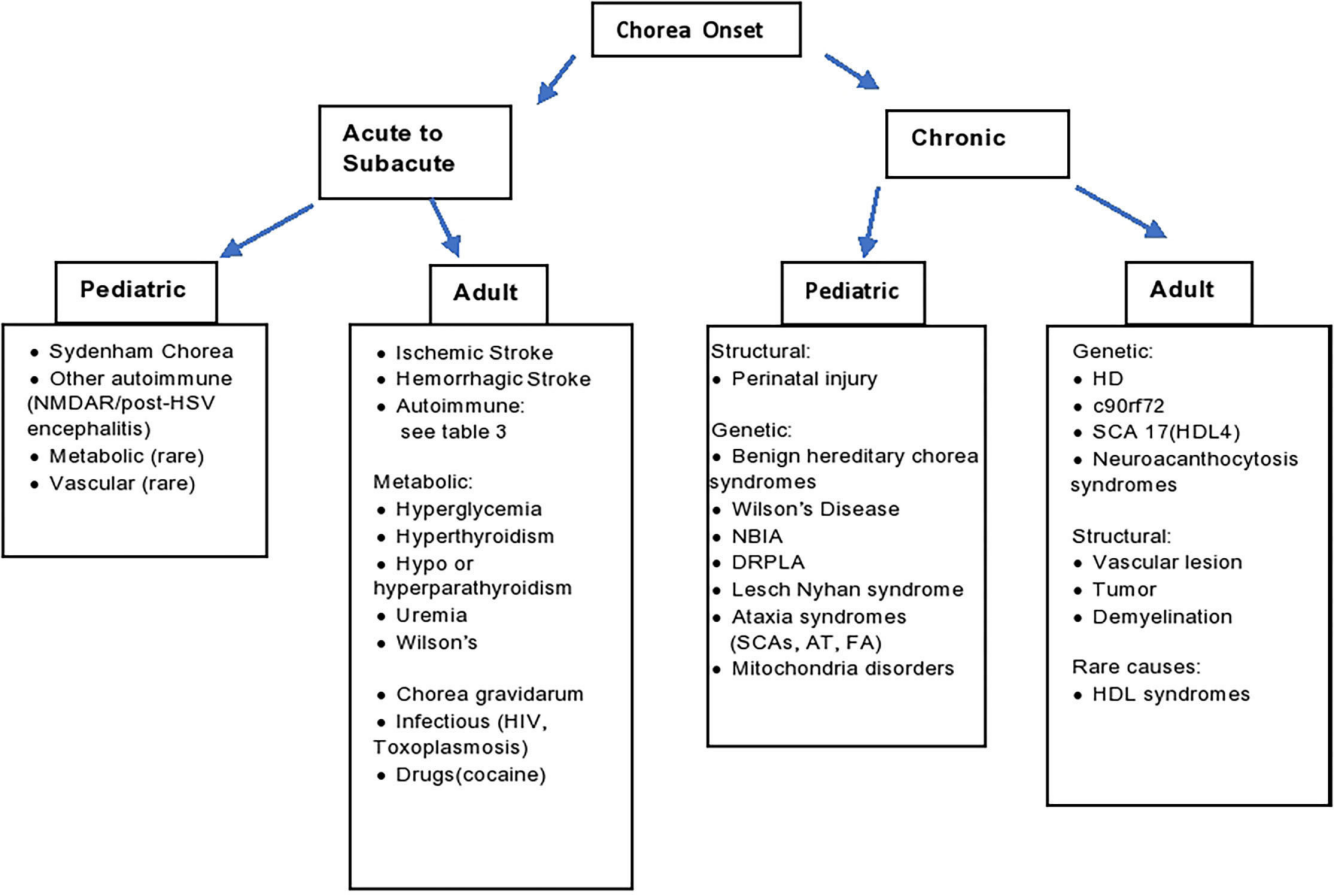
Diagnostic algorithm. AT, ataxia telangiectasia; DRPLA, dentatorubral-pallidoluysian atrophy; FA, Friedrich's ataxia; HD, Huntington's disease; HDL, Huntington's disease like; HIV, human immunodeficiency virus; HSV, herpes simplex encephalitis; NBIA, neurodegeneration with brain iron accumulation; SCA, spinocerebellar ataxia; SLE, systemic lupus erythematosus.

The appropriate work up for chorea should be guided by the clinical context, associated signs and symptoms, onset and temporal progression (3). With chronic symptomatology in adults one should, of course, consider genetic testing for HD, in addition to rarer genetic degenerative disorders. In children with a chronic, stable course and history of perinatal injury, cerebral palsy can be implicated. In children with a chronic, progressive course, a vast array of hereditary conditions can be considered (Figure 1), in which cases the diagnosis can be guided by associated phenotypic features such as ataxia, hypotonia, paroxysmal nature, amongst many others (4).

With acute onset, vascular etiologies should be considered. In the context of acute to subacute onset of chorea in a previously healthy child in the correct clinical context, an autoimmune phenomenon should be considered. The spectrum of autoimmune mediated chorea encompasses a heterogenous group of disorders including paraneoplastic, post-infectious and idiopathic (2, 5).

In the discussion of autoimmune chorea syndromes, it is important to be aware of the systemic associations and implications of each disorder diagnostically and prognostically. Sydenham's Chorea (SC), associated with streptococcal infections, for instance, warrants a thorough cardiac work up (2). Antibody mediated syndromes that are more likely to be paraneoplastic warrant thorough age-appropriate cancer investigations (1). Familiarity with the degree of immunotherapy responsiveness of each of the autoimmune disorders and overall prognosis is also of importance.

## Sydenham's Chorea

SC is a major feature of Rheumatic Fever that is observed in between 15 and 26% of patients with this disease (6, 7). SC is the most common cause of acute onset chorea in children and second most common cause of pediatric chorea after cerebral palsy, although the incidence of this disease has decreased, particularly in the Western world. It is a post-streptococcal autoimmune disease with latency of onset between 4 weeks to more than 6 months after group A beta hemolytic streptococcal (GABHS) infection (2, 7). The consensus postulation is that the autoimmune pathogenesis is mediated by molecular mimicry between the bacteria and neural antigens (8). The typical age of onset is 5–15 years. The clinical presentation is variable. Patients can present with generalized chorea, although ~20% exhibit hemichorea (5). Patients can also become hypotonic or develop motor impersistence, an inability to maintain voluntary contraction, which is a characteristic feature of chorea. In rare instances, the hypotonia can be profound enough to cause complete immobility, a condition termed chorea paralytica. There is growing interest in the neuropsychiatric manifestations including obsessive compulsive behavior, decreased verbal fluency and executive dysfunction as potential manifestations of the same autoimmune process (2, 9). Chorea is typically observed before the onset of these neuropsychiatric features. Long term neuropsychiatric manifestations have been observed in up to 20% of cases (10).

SC is one of the major diagnostic components in the Jones Criteria for rheumatic fever, along with carditis, arthritis, erythema marginatum, and subcutaneous nodules (11). In terms of proving recent GABHS infection, antistreptolysin O (ASO) antibodies may not be discovered due to the latency in onset of chorea, although anti-DNase antibodies may still be identifiable. Neuroimaging is usually normal, however, there are rare reports of increased basal ganglia structure size on volumetric analysis and reversible T2 hyperintensity on MRI (12–16).

In terms of symptomatic treatments, the efficacy of tetrabenazine, a vesicular monoamine transport 2 (VMAT-2) inhibitor, has been demonstrated by open label studies (17). Valproic acid and carbamazepine have had reported efficacy in case series (18). Neuroleptics are second line, given the risk of extrapyramidal side effects (19). For patients with ongoing refractory chorea, immunotherapy with IV methylprednisolone has been reported to be helpful (20, 21). One small randomized control trial demonstrated improvement in chorea after 1 month with either IVIg, plasma exchange or steroids (22). The observed response to immunotherapy provides further support for an autoimmune etiology (22, 23).

The need for cardiac monitoring should be emphasized, as up to 60% of patients with chorea can have residual rheumatic heart disease (7). Secondary prophylaxis with continuous benzathine penicillin or a macrolide antibiotic, in cases of penicillin allergy, is recommended to mitigate the risk of recurrence rheumatic fever (24). The duration of prophylactic antibiotic use is dependent on underlying factors, ranging from 5 years or until age 21 in patients without carditis; 10 years or age 21 in patients with carditis, without residual cardiac disease; 10 years or until age 40 in carditis patients with residual cardiac disease (24).

In terms of prognosis, some studies have demonstrated ongoing chorea in 50% of patients at 2-year follow-up. Risk of chorea recurrence is said to be around 30%, despite continuous antibiotic prophylaxis (25, 26). Adulthood recurrence was more common during pregnancy, as Chorea Gravidarum, in a cohort of patients with initial onset at a median age of 12 years (27). Additionally, the study reported that patients with Chorea Gravidarum that were subsequently treated with oral contraceptives all had chorea recurrence. This suggests a susceptibility in patients with a history of SC to increased estrogen, potentially due to the sensitization of basal ganglia dopamine receptors.

There is an ongoing effort to identify whether specific neural antibodies mediate the chorea in SC. As yet there is no definite autoantigen, though it has been shown that antibodies from SC patients cross react with GABHS cell wall carbohydrate and CNS gangliosides (8). There has been some evidence for the role of dopaminergic pathways, with D1 and D2 antibodies in rodent models demonstrating similar clinical features (28, 29). Several studies have described neuronal surface binding and neuronal tubulin binding antibodies inducing a calcium/calmodulin-dependent protein kinase 2 that increases dopamine release (8, 30). Overall, it has been reported that basal ganglia-targeted antibodies have been discovered in up to 90% of patients (2, 31). They do not appear to be specific to the basal ganglia, however, as these antibodies have also been identified in healthy patients, as well as in patients with Huntington's and Parkinson's Diseases (32). Thus, there is still debate regarding the precise role of these antibodies in neuronal dysfunction and chorea, which may become clearer with future studies.

## Paraneoplastic

The association of chorea with cancer is extremely rare in children. In adults, there is a continuously expanding list of antibodies implicated in paraneoplastic chorea, as outlined in Table 1. As is typical for paraneoplastic neurologic syndromes, chorea can predate the discovery of the neoplasm for months to years (1, 2). Chorea often manifests in the context of other movement disorders and encephalopathy. The most common associated neoplasm is small cell lung carcinoma (SCLC). The most commonly identified antibodies in paraneoplastic mediated chorea are CRMP-5 (CV2) and ANNA-1 (Hu) IgG. About 70% of CRMP-5-associated chorea is associated with SCLC, followed by thymoma in 30% (33, 34). Less commonly, CRMP-5 chorea has been reported with non-Hodgkin's lymphoma and tonsillar squamous cell carcinoma (35). More recently a new antibody, phosphodiesterase 10A IgG (PDE10A) was identified as a biomarker of paraneoplastic neurologic autoimmunity (36). Half (3/7) of patients with serum PDE10A antibodies had chorea or ballismus. Two of these patients had the onset of movement disorders after the use of immune checkpoint inhibitor [ICI] therapy, with notable T2 FLAIR hyperintensities in the basal ganglia. The association with ICI therapy, a relatively novel approach in oncology, is pertinent to note. There is likely to be an increased frequency of immune mediated chorea as the use of ICI therapy expands. Autoimmunity associated with ICI is postulated to be related to inhibition of the regulatory steps of T-cell immune mechanisms, thereby potentially increasing the propensity for aberrant T cell activation and immune mediated adverse events (37). Among the PDE 10A chorea patients one had a lung nodule, one had renal cell cancer and one had lung carcinoma.

**Table 1:** Summary of clinical syndromes and antibodies.

**Syndrome**	**Clinical features**	**Antibodies**	**Treatment**	**Comments**
Parainfectious: Sydenham's Chorea	- Major feature of Rheumatic Fever - (SC in 15–26% of cases) - Typical onset 5–15 yr - Recurrence in adulthood (pregnancy/hormones) - Chorea: generalized or hemichorea; chorea paralytica - Psychiatric: Tics, OCD, verbal fluency, executive dysfunction	Neuronal surface antibodies (pathogenicity of these debated): - D1/D2 - Lysoganglioside - Neuronal tubulin	Symptomatic: - VMAT 2 inhibitors - VPA/CBZ - Neuroleptic Refractory Cases: - IVMP, IVIG, PLEX - Penicillin prophylaxis	- Perform cardiac work up (up to 60% have residual cardiac disease) - ASO antibodies may not be seen given latency between infection and onset of chorea - Anti-DNase may be still be identified
Paraneoplastic: CRMP-5/CV2	- Most commonly identified paraneoplastic chorea - Associated with SCLC (70%), thymoma (30%), rarely Non-Hodgkin lymphoma, tonsillar SCC - Comorbid orobuccal dyskinesia, dystonia, polyneuropathy	CRMP-5 (CV2) (intracellular)	Oncologic Immunotherapy: - IVMP, IVIg, PLEX - Cyclophosphamide (Cyc) Symptomatic: - VMAT 2 inhibitors - VPA/CBZ - Neuroleptic	- Perform neoplastic work up - Antibody to intraneuronal antigen; not pathogenic - Poor prognosis - Rarely seen in pseudoathetosis (secondary to sensory neuronopathy)
Paraneoplastic: ANNA-1/Hu	−2nd most common antibody seen in paraneoplastic chorea - Most commonly associated with SCLC	ANNA-1 (Hu) (intracellular)	See CRMP-5/CV2	- See CRMP-5/CV2 - Most common paraneoplastic cause of pseudoathetosis.
Idiopathic or paraneoplastic: LGI1, CASPR-2, GAD-65	- All cause Limbic Encephalitis - More commonly associated with other autoimmune neurologic phenomena - (GAD-65: stiff person, cerebellar ataxia, seizures; LGI1: faciobrachial dystonic seizures; CASPR2: Isaac's/Morvan's syndromes)	LGI1, CASPR2 (cell surface) GAD-65 (intracellular)	- IVMP, IVIg, PLEX - 2nd line immunotherapies: RTX, Cyc	Consider cancer work up (cancer related cases have been described).
Idiopathic, post-infectious, or paraneoplastic: NMDAR	- Median age 21 - F: M ratio = 4:1 - Most common sporadic encephalitis. - 1st phase: Psychiatric, sleep disturbance - 2nd phase: Movement disorders—dyskinesia, stereotypy, dystonia, chorea. - Teratoma in ~50% adult females. - Tumors are rare in M and F <14	NMDAR Glu N1 Subunit (cell surface)	−1st line: IVMP, IVIg, PLEX - 2nd line: RTX or Cyc - 3rd line: tocilizumab or bortezomib	- Autoimmune/paraneoplastic: similar presentation - Post HSV NMDAR encephalitis often manifests with choreoathetosis in children
Idiopathic and/or neurodegenerative: IgLON-5 disease	- Median age 64 - Parasomnias - Chorea (35%) - Cognitive decline - Periodic limb movements. - Brainstem involvement: bulbar symptoms, eye movement abnormalities, stridor, sudden death in sleep.	IgLON5 (cell surface; neuronal cell adhesion protein)	Increased evidence of immunotherapy response if treated early: - IVMP, IVIg, PLEX - Cyc, RTX - AZA, MMF	- Tau aggregates in brainstem/ tegmentum and hypothalamus - Hypothalamic dysfunction - Debate regarding neurodegeneration or autoimmune as primary pathology
Systemic Disease: SLE/PAPS	- Median age of onset 15–26 - F > 90% of cases - Chorea 1–2% of SLE/PAPS patients - Chorea may precede onset of SLE - OCP/Pregnancy increases risk	aPL antibodies; - Beta-2 Glycoprotein - Anticardiolipin - Lupus anticoagulant SLE antibodies: - ANA - dsDNA - RNP, Smith	Symptomatic: - VPA/CBZ - VMAT2 inhibitors - Neuroleptic Refractory or systemic symptoms: - IVMP, IVIg, PLEX - Cyc Anticoagulation in 1° or 2°	Possible antibody mechanisms: - Vascular - Blood brain barrier cytokine - NMDAR NR2A/3B

*ANA, antinuclear antibody; ANNA-1, antineuronal nuclear antibodies type 1; aPL, phospholipid antibody; ASO, antistreptolysin; AZA, azathioprine; CASPR-2, contactin-associated protein-2; CBZ, carbamazepine; CRMP-5, collapsin response-mediator protein 5; Cyc, cyclophosphamide; GABHS, Group A Beta Hemolytic Streptococcus; GAD-65, glutamic acid decarboxylase 65-kDa isoform; IVIg, intravenous immunoglobulin; IVMP, intravenous methylprednisolone; LGI1, leucine-rich glioma-inactivated 1; MMF, Mycophenolate mofetil; NMDAR, N-Methyl-D-aspartic acid receptor; OCP, oral contraceptive pill; PAPS, primary antiphospholipid syndrome; PLEX, plasma exchange; RTX, rituximab; SLE, systemic lupus erythematosus; VMAT-2, vesicular monoamine oxidase-2 inhibitor; VPA, valproic acid*.

A cohort of 13 paraneoplastic chorea patients was described in 2011, with most presenting with symmetric bilateral chorea in addition to other neurologic manifestations, including dystonia, peripheral neuropathy, encephalopathy and/or psychiatric manifestations (38). The prognosis for both the treatment of chorea and overall survival in patients with CRMP-5 and ANNA-1-related disease is poor. The largest single site analysis of a cohort of autoimmune chorea patients found that 14/36 [39%] identified cases were paraneoplastic (1). The median age of onset of chorea in this group was 72. Seventy-one percent of the patients were male. The most common neoplasm was SCLC, followed by non-small cell lung carcinoma, hematologic malignancy and various adenocarcinomas. Cancer predictive antibodies included CRMP-5 (4 patients: 1 case of tonsillar squamous cell, 2 mixed histology lung carcinomas and 1 SCLC), ANNA-1 (3 patients: 2 with lung carcinoma and 1 bladder carcinoma), followed by ANNA-2 (Ri) and amphiphysin antibodies in a patient with SCLC. Improvement in chorea was recorded in 3/7 [43%] patients in relation to oncologic therapy, in 5/11 [45%] patients in relation to immunotherapy, and in 6/12 [50%] patients from symptomatic therapies. Immunotherapies included steroids, plasma exchange, cyclophosphamide and steroids with IVIg.

Identifying and treating the underlying cancer are of course, pivotal, although it does not consistently ensure symptom resolution. Currently there are no randomized or larger clinical trials delineating optimal immunomodulatory treatment algorithms in paraneoplastic neurologic disease. Thus, immunotherapy approaches are drawn from expert opinion, extrapolated from paraneoplastic encephalitidies in general (1, 35, 39, 40). Treatment may include IV methylprednisolone and cyclophosphamide; however, syndromes associated with intracellularly targeted antibodies tend to be refractory to treatment (1, 40).

## Idiopathic Autoimmune Chorea

Non-paraneoplastic, antibody-positive chorea has been reported in association with multiple neuronal antibodies thus far including GAD-65, CASPR2, and LGI1, voltage gated calcium channel antibody (VGCC) and striational antibodies, as outlined in Table 1 (1, 41, 42). It should be emphasized that chorea is a rare manifestation of these antineuronal antibody syndromes, particularly in isolation. Neurologically, high titer GAD-65 autoantibodies are more often associated with stiff person syndrome, cerebellar ataxia, limbic encephalitis, and epilepsy (43). GAD-65 antibodies, usually at low titers, are also associated other autoimmune disorders such type 1 diabetes, pernicious anemia, and autoimmune thyroiditis (44). It is recognized that syndromes previously ascribed to anti-voltage gated potassium channel (VGKC) antibodies are actually mediated by more specific antibodies against proteins associated with the potassium channel complex, LGI1 and CASPR2 (45). LGI1 antibodies are the second most common cause of limbic encephalitis. LGI1 and CASPR2 antibodies are felt to be pathogenic, and are associated with well-described syndromes that typically do not include chorea (46). LGI1 antibodies are associated with faciobrachial dystonic seizures, limbic encephalitis, and seizures (47). CASPR2 antibodies are associated with Isaac's syndrome of neuromyotonia; Morvan's, a syndrome of neuromyotonia, dysautonomia and encephalopathy; and seizures (46, 48). These antibodies are also implicated in paraneoplastic disease, although less commonly for LGI1. For instance, CASPR2 positivity in Morvan's Syndrome is associated with thymoma (48).

In the cohort of autoimmune chorea patients reported by O'Toole et al. 22/36 [61%] cases were non-paraneoplastic (1). The median age of onset was 45. 14 cases exhibited focal or segmental chorea. There were additional neurologic symptoms in 14 patients, including cognitive and behavioral or peripheral neuropathy manifestations. Neuronal antibodies included GAD-65 in 3 cases, P/Q-VGCC in 2, N-VGCC in 1, ACh-R in 1, striational in 2 and CASPR2 in 1. The GAD antibody titer in at least 1 patient was high titer, at 760 nmol/l. Similarly, the CASPR2 antibody was high titer. These patients were treated with steroids alone or with IVIg and azathioprine. 7 [32%] patients had near complete remission. Spontaneous improvement, or remission without treatment, was seen in 13 [59%] patients.

## NMDA Receptor Encephalitis (NMDARE)

Chorea is a rare manifestation of the complex neurological syndrome of NMDARE, the most common sporadic autoimmune encephalitis, though a variety of involuntary movements are commonly seen, particularly in children. The NMDAR antibody was first reported in 2007 (49). NMDAR antibodies target the N-terminal end of the GluN1 subunit of the NMDAR. The consensus is that NMDAR antibodies are pathogenic (50–52). This disease most commonly occurs in children and younger adults. The median age of onset is 21. The ratio of females to males is 4 to 1 (53, 54). The annual incidence is 1.5 per million (54). It can be paraneoplastic, post-infectious, or idiopathic in etiology. Ovarian teratomas are identified in ~1/2 of adult women and 1/3 of teenage females. Paraneoplastic disease is rarely seen in males and female children younger than 14 (50). Additional cancers identified in older adults with paraneoplastic disease include lung, breast, testis, pancreas, thymic, and Merkel Cell carcinoma (53, 55).

Patients can experience an initial viral-like prodrome followed by a phase of neuropsychiatric symptoms characterized by psychosis, disinhibition, sleep disorders, catatonia and seizures. If untreated, the neuropsychiatric phase progresses and patients can express a myriad of movement disorders including a combination of stereotypies, dystonia, myorhythmia, blepharospasm, ataxia and chorea (2). Over 75% of patients develop movement disorders (50, 54, 56, 57). However, true chorea is described in a minority of patients (54, 58). For instance, the orobuccal movements are more commonly described as dyskinetic rather than chorea, in that they are more stereotyped in nature, or they can occasionally represent myorhythmia. The diagnosis is based on the identification of NMDAR antibodies, which are more specific when identified in the CSF than serum (50). Approximately 1/3 of patients will have an abnormal MRI, with increased T2 signal in a non-specific pattern cortically and/or subcortically (50).

There is increasing recognition of a phenomenon of secondary autoimmune encephalitis with demonstrable NMDAR antibodies occurring after HSV encephalitis (59–61). Antibodies are typically detected 1–4 weeks after initial infection. Autoimmune encephalitis has been reported in up to 27% of patients after initial HSV infection (62). Interestingly, in contrast to adults, choreoathetosis is a prominent and severe manifestation in younger children with post-HSV NMDARE (63). In addition, recovery at 1 year seems to be worse than typical NMDAR encephalitis, particularly in children (63). This has been treated in a similar manner to NMDAR encephalitis, with immunotherapy.

Guidance for immunotherapy comes from observational cohorts and expert opinion. First line treatment consists of steroids, IVIg or plasmapheresis, or a combination of these modalities (64). Second line agents include rituximab or cyclophosphamide (53). Further biologic therapy such as tocilizumab (an interleukin-6 receptor inhibitor) or bortezomib (a proteasome inhibitor) have been utilized in refractory cases. The established consensus is that earlier treatment portends better prognosis (56). The response rate to immune therapy is reported to be ~80% (53). Overall, there is a 12% recurrence rate (50, 56, 65). The estimated mortality rate was previously quoted to be 4%, prior to more broad recognition of this disorder (66).

## Anti-IgLON5 Disease

IgLON5-associated disease is a more recently described entity. Interestingly, distinct from the other autoimmune disorders discussed, neurodegeneration has been documented on pathologic studies (67–70). This disorder is characterized by chorea in up to 35% of patients, as well as REM and non-REM parasomnias, dysautonomia, brainstem and hypothalamic dysfunction. Another common presentation resembles progressive supranuclear palsy, with vertical gaze palsy. Additional features include periodic limb movements in wakefulness and early sleep, in addition to stridor and sudden death in sleep or wakefulness (67). The implicated antibody targets IgLON5, a neuronal cell adhesion protein. The median age of onset is 64 and the most common presentation in a case series of 22 patients was cognitive decline and chorea (69). A more recent case report described a typical IgLON5 syndrome with sleep disturbance and bulbar syndrome, antibody positivity in serum and CSF, but interestingly without phosphorylated tau in the brainstem, as had been reported in the original case series of IgLON5 patients (70). The patient did not respond to immune therapy. Indeed, motor neuron disease has been recently identified as an associated phenotype with IgLON5 autoantibodies (71, 72).

Neuropathology findings in IgLON5-associated disease were published as part of consensus criteria in 2016 (68). The authors outlined the pertinent findings including neuronal accumulation of hyperphosphorylated tau, with both three-repeat (3R) and four-repeat (4R) tau isoforms, involving the hypothalamus, and more severely the tegmental nuclei of the brainstem, with a cranio-caudal gradient of severity to the upper cervical cord (68). The initially reported features of progressive decline over a longer period of time (>1 year), poor response to immunotherapy, and documented neurodegeneration collectively gave rise to controversy as to whether autoimmunity or neurodegeneration is the primary overarching pathology. More recently, however, reports have illustrated responsiveness to immunotherapy with early treatment, with systematic review demonstrating response to immunotherapy in 20/46(43%) of patients included. Non-classical phenotype, cognitive impairment, evidence of CSF inflammation, certain haplotypes, combination immunotherapy, and use of second line immunotherapy all seemed to be associated with treatment responsiveness (73, 74).

## SLE and Primary Antiphospholipid Syndrome

Chorea manifests in SLE or PAPS patients in ~1–2% of cases (75, 76). Other neuropsychiatric features are more common than movement disorders in SLE, although chorea is the most common movement disorder. Chorea may precede the onset of SLE in over 20% of cases (75). There is a female predominance in over 90% of cases (77). The typical age of onset is between 15 and 26 years of age (75). Oral contraceptive use and pregnancy are risk factors for developing chorea in these disorders; both SLE and PAPS are well recognized causes of chorea gravidarum (27, 77, 78). In terms of clinical patterns, a case series of 50 patients with SLE/PAPS chorea found that 55% had unilateral presentation (77). Sixty-six percent of cases were monophasic and the remainder experienced relapses.

The pathogenesis of chorea in SLE/PAPS is still not well-understood. In terms of functional imaging, FDG-PET studies in SLE and antiphospholipid syndrome patients have revealed increased metabolism in the contralateral striatum (79–82). There is a high incidence of antiphospholipid antibodies (aPL) in studies of SLE patients with neuropsychiatric manifestations, between 25 and 90%, perhaps implicating a role of these antibodies in the pathogenesis (83–85). This is further bolstered by the incidence of chorea in PAPS. The cohort described by O'Toole et al., of 36 autoimmune chorea patients, revealed 50% of cases had aPL antibodies. There were eight patients with SLE, six of whom had concomitant antiphospholipid syndrome and two cases of PAPS (1). There is ongoing research into whether these antibodies contribute pathogenicity. One purported mechanism of action of this antibody is vascular, qualified clinically by the incidence of thrombotic events in patients (86). Other potential theories include NMDAR antibodies against NR2A/B subunits, causing direct neuronal dysfunction, which is observed in SLE with psychiatric symptoms, but not chorea specifically (75, 87, 88). An additional possible mechanism is disruption of the blood brain barrier with CNS infiltration of inflammatory cytokines, causing damage and cell death (2, 85).

With regards to symptomatic treatment of SLE/PAPS-associated chorea, the use of VMAT-2 inhibitors, valproic acid, carbamazepine and neuroleptics have all been described (75). Refractory cases and patients with additional systemic manifestations have been treated with immunotherapy including steroids, IVIg, plasmapheresis and cyclophosphamide (89, 90). In the aforementioned cohort of 36 autoimmune chorea patients, SLE and PAPS chorea patients were universally steroid responsive (1). Patients with true primary or secondary antiphospholipid syndrome are recommended to be treated with oral anticoagulants, due to the relatively high incidence of thrombosis in these patients. Interestingly, no basal ganglia ischemia was found in the patients described by O'Toole et al.

## Approach to Diagnosis

As outlined in Figure 1, the approach to diagnosis is guided by several factors, including age, acuteness of onset, and ultimately associated symptoms. In the context of chronic symptomatology in adults, one should consider genetic etiologies, particularly HD, as well as the HD phenocopies (SCA 17, C9Orf 72) and neuroacanthocytosis. Structural lesions of the basal ganglia should of course be excluded. Chronic symptoms in a child are most often underpinned by perinatal injury or genetic causes (such as Benign Hereditary Chorea, Wilson's disease, Spinocerebellar Ataxias, Dentatorubral-Pallidoluysian Atrophy and mitochondrial disorders). In hyperacute to acute situations, vascular etiologies should be suspected. In acute to subacute presentations, one should also exclude metabolic, endocrine etiologies such as hyperglycemia basal ganglia syndrome. Additionally, we should be mindful about pseudoathetosis as a mimic with similar phenomenology, with a broad range of etiologies beyond the scope of this review.

Within the acute to subacute window of onset, one should consider autoimmune etiologies. In children, SC is by far the most common etiology, though interestingly, NMDAR has been observed to manifest with chorea in children, particularly after HSV encephalitis. Paraneoplastic chorea is exceedingly uncommon in children. In adults, we can examine for associated symptoms to help delineate specific autoimmune and paraneoplastic syndromes, as summarized in Table 2 and elaborated in Table 1.

**Table 2:** Autoimmune causes of chorea.

**Paraneoplastic (in order of prevalence)**
• CRMP-5(CV2)
• ANNA-1(Hu)
• NMDAR
• Uncommon: ANNA-2(Ri), CASPR2, PDE-10A
**Systemic disease**
• SLE
• PAPS
**Idiopathic autoimmune**
• NMDAR
• GAD-65
• CASPR2
• LGI1
**Idiopathic autoimmune and/or neurodegenerative**
• IgLON5

*ANNA, antineuronal nuclear antibodies; CASPR-2, contactin-associated protein-2; CRMP-5, collapsin response-mediator protein 5; GAD-65, glutamic acid decarboxylase 65-kDa isoform; IgLON5, immunoglobulin-like cell adhesion molecule 5; LGI1, leucine-rich glioma-inactivated 1; NMDAR, N-Methyl-D-aspartic acid receptor; PAPS, primary antiphospholipid syndrome; PDE-10A, phosphodiesterase 10-A; SLE, systemic lupus erythematosus*.

## Conclusion

Autoimmune chorea encompasses a heterogenous set of disorders spanning parainfectious, paraneoplastic, systemic, and idiopathic diseases. The approach to diagnosis should be guided by the clinical context including age, associated features, onset and chronicity of symptoms.

Acute to subacute onset of chorea in children should give rise to a clinical suspicion of autoimmune chorea, with SC being the most common cause. There is ongoing research to elucidate specific antineuronal antibodies implicated in the pathogenesis. Additional autoimmune causes of chorea in children, such as anti-NMDAR should be considered in the appropriate context. NMDAR antibody syndrome typically causes orobuccal dyskinesia, which is stereotypic, rather than true chorea, although post-HSV NMDARE cases seem to manifest with choreoathetosis more commonly. Paraneoplastic causes of chorea in children are exquisitely rare.

In adults, both paraneoplastic and idiopathic autoimmune chorea are observed.

Paraneoplastic chorea in older adults is most commonly associated with intracellular antigens, such as CRMP-5, followed by ANNA-1 antibodies, in association with small cell lung carcinoma. Other paraneoplastic antibodies and cancers are described, but less commonly. Responsiveness to immune therapy is variable, from the limited data available, but the prognosis is generally poor in paraneoplastic disease.

Presentations of idiopathic autoimmune chorea with neuronal specific antibodies include GAD-65, LGI1, and CASPR2, although these antibodies are not specific for chorea and are associated with a myriad of other neurologic signs. There is evidence that these syndromes are responsive to immune therapy. Chorea can also manifest in the context of systemic autoimmune illnesses, including SLE and PAPS. Interestingly, in SLE patients exhibiting chorea, there is a high incidence of antiphospholipid antibodies.

The more recently described entity of IgLON5 disease requires further investigation to better understand the etiology, recognizing that although there is a consistently identified antibody against IgLON5, there is evidence of a neurodegenerative process as well. Interestingly, recent reports describe response to immunotherapy in cases that are identified earlier. This perhaps raises the questions of whether the initial reports identified later onset cases, at a more predominantly neurodegenerative stage of disease, and whether there is a spectrum between autoimmune and neurodegenerative disease.

The recent discovery of yet another biomarker of paraneoplastic associated autoimmune chorea, the PDE10A antibody and the association with ICI neurologic autoimmunity underscores that there is an ongoing imperative need to discover further novel antibodies and to investigate their potential pathogenicity in autoimmune chorea. This knowledge could ultimately aid diagnostic confidence, guide investigative decisions and treatment and ultimately improve patient outcomes.

## Author Contributions

KK contributed to conception, organization, and writing of first draft of this review. YB, NV, and JL contributed to manuscript critique and revision. All authors read and approved the final manuscript.

## Conflict of Interest

The authors declare that the research was conducted in the absence of any commercial or financial relationships that could be construed as a potential conflict of interest.

## Publisher's Note

All claims expressed in this article are solely those of the authors and do not necessarily represent those of their affiliated organizations, or those of the publisher, the editors and the reviewers. Any product that may be evaluated in this article, or claim that may be made by its manufacturer, is not guaranteed or endorsed by the publisher.
